# Glucosinolate Bioactivation by *Apis mellifera* Workers and Its Impact on *Nosema ceranae* Infection at the Colony Level

**DOI:** 10.3390/biom11111657

**Published:** 2021-11-08

**Authors:** Luisa Ugolini, Giovanni Cilia, Eleonora Pagnotta, Lorena Malaguti, Vittorio Capano, Irene Guerra, Laura Zavatta, Sergio Albertazzi, Roberto Matteo, Luca Lazzeri, Laura Righetti, Antonio Nanetti

**Affiliations:** 1 Research Centre for Cereal and Industrial Crops (CREA-CI), Council for Agricultural Research and Agricultural Economics Analysis, Via di Corticella 133, 40128 Bologna, Italy; luisa.ugolini@crea.gov.it (L.U.); lorena.malaguti@crea.gov.it (L.M.); roberto.matteo@crea.gov.it (R.M.); luca.lazzeri@crea.gov.it (L.L.); laura.righetti@crea.gov.it (L.R.); 2 Research Centre for Agriculture and Environment (CREA-AA), Council for Agricultural Research and Agricultural Economics Analysis, Via di Saliceto 80, 40128 Bologna, Italy; giovanni.cilia@crea.gov.it (G.C.); vittorio.capano@crea.gov.it (V.C.); irene.guerra@crea.gov.it (I.G.); laura.zavatta@crea.gov.it (L.Z.); sergio.albertazzi@crea.gov.it (S.A.); antonio.nanetti@crea.gov.it (A.N.)

**Keywords:** Brassicaceae, *Brassica nigra*, *Eruca sativa*, nosemosis, honey bee, field trials, isothiocyanate, myrosinase, honey

## Abstract

The microsporidian fungus *Nosema ceranae* represents one of the primary bee infection threats worldwide and the antibiotic fumagillin is the only registered product for nosemosis disease control, while few alternatives are, at present, available. Natural bioactive compounds deriving from the glucosinolate–myrosinase system (GSL–MYR) in Brassicaceae plants, mainly isothiocyanates (ITCs), are known for their antimicrobial activity against numerous pathogens and for their health-protective effects in humans. This work explored the use of *Brassica nigra* and *Eruca sativa* defatted seed meal (DSM) GSL-containing diets against natural *Nosema* infection in *Apis mellifera* colonies. DSM patties from each plant species were obtained by adding DSMs to sugar candy at the concentration of 4% (*w*/*w*). The feeding was administered in May to mildly *N. ceranae*-infected honey bee colonies for four weeks at the dose of 250 g/week. In the treated groups, no significant effects on colony development and bee mortality were observed compared to the negative controls. The *N. ceranae* abundance showed a slight but significant decrease. Furthermore, the GSL metabolism in bees was investigated, and MYR hydrolytic activity was qualitatively searched in isolated bee midgut and hindgut. Interestingly, MYR activity was detected both in the bees fed DSMs and in the control group where the bees did not receive DSMs. In parallel, ITCs were found in gut tissues from the bees treated with DSMs, corroborating the presence of a MYR-like enzyme capable of hydrolyzing ingested GSLs. On the other hand, GSLs and other GSL hydrolysis products other than ITCs, such as nitriles, were found in honey produced by the treated bees, potentially increasing the health value of the final product for human consumption. The results are indicative of a specific effect on the *N. ceranae* infection in managed honey bee colonies depending on the GSL activation within the target organ.

## 1. Introduction

The decline of the honey bee population and the worrying threats to their survival are currently dominating the public debate. Toxic pesticides, intensive agriculture, land urbanization, climate change and diseases are the main reasons, often acting in synergy, of the bee numbers decline across the world. Among the bee parasites and pathogens linked to colony decline, *Nosema ceranae* represents one of the primary bee infection threats [1].

*N. ceranae*, a microsporidian fungus first identified in the Asian honey bee *Apis cerana*, has replaced *Nosema apis* as a parasite of the European honey bee *Apis mellifera*, and it is rapidly spreading around the world [2,3]. The microsporidian has a tropism for the honey bee midgut epithelial cells [4], and its infection causes alteration of the homeostasis and renewal of intestinal tissues and consequent malnutrition, impaired behavior orientation, suppressed immune function and final colony losses [5,6,7].

To date, the only registered product for *N. ceranae* control is the antibiotic fumagillin, a compound toxic to mammals, the effectiveness studies whereof often provided contradictory results, mostly because its use poses health risks concerning pathogen resistance insurgence and bee products contamination [8,9,10]. Therefore, there is an increasing demand for safer and effective treatments for *N. ceranae*, and a variety of natural compounds have been investigated in laboratory and field trials [11]. For instance, the role and functions of probiotics in honey bee health have recently been gaining great scientific interest [12,13,14]. Supplemented microorganisms, such as *Bifidobacterium* and *Lactobacillus* strains, known for secreting antibiotic metabolites exhibited a positive effect in controlling the infection, lowering *N. ceranae* spore levels [14]. The commercial probiotic Protexin^®^ (*Enterococcus faecium*) showed promising results for *N. ceranae* control and honey bee population promotion both in laboratory and field trials [9,15]. Nevertheless, the interaction of gut microbiota and microsporidia is still not completely understood, and further research is needed to clarify probiotic mechanisms of action [16]. Among the fumagillin alternatives, dietary supplements based on vitamins, plant extracts and phytochemicals appeared to be promising in the control of nosemosis in several studies [17,18,19,20,21,22,23]. In this context, natural products deriving from plants of the Brassicaceae family have been intensively studied for their antimicrobial properties and health benefits in animals and humans. Bioactive compounds are produced in Brassicaceae plant tissues by the endogenous defensive glucosinolate–myrosinase (GSL–MYR) system upon tissue disruption and subsequent GSL hydrolysis by the enzyme MYR [24,25]. The hydrolysis reaction could lead to different products, isothiocyanates (ITCs), nitriles, epithionitriles, hydroxynitriles, oxazolidine-2-thiones or thiocyanate, depending on the GSL substrate structure, environmental conditions, and the presence of specifier proteins [26]. GSLs are a structurally diverse group of β-thioglucoside-N-hydroxysulfates with a variable hydrophobic aglucone side chain (R-group). The GSL R-groups are maintained in the hydrolysis products determining their physicochemical and biological properties and their classification in aliphatic, aryl aliphatic, or indolic in the function of the side chain structure [27,28,29].

MYR is a β-thioglucosidase (E.C. 3.2.1.147) belonging to glycoside hydrolase family 1 (GH1), found in the myrosin cells sequestered from the cells rich in GSL-containing vacuoles. The most characterized plant MYR isoenzyme was isolated from ripe seeds of white mustard, *Sinapis alba* [30,31], but MYR has been identified in brassica specialist aphids [32] and insects, too, such as the specialist *Phyllotreta striolata* flea beetles [33,34]. Anyway, plant, aphid and insect MYR showed to share a low level of sequence identity and probably hold some different biochemical characteristics, such as the different susceptibility to ascorbic acid [33]. MYR is also present in human gut microbiota that is partly responsible for the beneficial effect of Brassicaceae vegetable consumption as bacteria metabolize GSLs, introduced with the diet, in the biologically active ITCs [35]. A bacterial MYR was isolated and for the first time completely gene-sequenced from a soil bacterium, *Citrobacter* Wye1, and belonged to the GH3 family of β-O-glucosidases, showing very little homology with plant or aphid MYR [36]. Besides, the importance of the gut microbiome in insect health and its role in the detoxification of plant toxic compounds has been less studied, but it has recently been gaining more and more attention [37].

ITCs are the main GSL hydrolysis products formed at neutral pH by the action of MYR and are compounds largely known for their broad-spectrum biological activities against pests and soil/food-borne fungi, bacteria and human microorganisms [29,38,39,40,41]. Their biological activity is a consequence of their chemical reactivity due to the presence of a very electrophilic carbon atom that attacks thiol, amine and alcohol groups in amino acids and proteins, impairing their function [42]. One of the main targets of ITCs is also the intracellular glutathione (GSH) pool, and in fungi, ITCs exert their biological activity by affecting redox homeostasis and inducing oxidative stress [43]. ITCs have also been largely studied for their beneficial effects on human health as they exhibit protection against neurodegenerative disorders, cancer and inflammatory diseases. They are strong inducers of phase II detoxification enzymes involved in the elimination of reactive anti-oxidants (ROS) and also induce cell cycle arrest and apoptosis in cancer cells [44,45,46].

The GSL–MYR system is particularly concentrated in Brassicaceae seeds, and the defatted seed meal (DSM), as a low-cost byproduct of oil extraction, represents a natural product also rich in other phytochemicals with high health-promoting benefits such as vitamin C, polyphenols and minerals [47]. For this reason, DSMs are exploited for different applications in the agri-food sector [48,49,50,51,52,53].

In laboratory trials, Borges et al. [54] studied the effect of the pure compound D,L-sulforaphane, the ITC product of the GSL glucoraphanin, on artificially *N. ceranae*-infected bees, finding a consistent reduction in the pathogen spores, but also high toxicity of the compound at higher doses. Instead, the use of DSMs of *Brassica nigra* and *Eruca sativa* showed to contain artificial *N. ceranae* infection and protect the bees at low doses, extending their lifespan [55]. ITCs were also found in the bee midgut and hindgut, suggesting the presence of a MYR-like activity able to metabolize GSLs from the DSMs in those tissues. However, this Brassicaceae biomasses have not yet been tested in field studies.

This study aimed to explore the effect of *B. nigra* and *E. sativa* DSM GSL-containing diets against natural *N. ceranae* infection on the colonies of *A. mellifera* in field studies. The GSL metabolism by MYR activity in the bee gut and the presence of GSLs and GSL-derived hydrolysis compound residues in honey was also investigated.

## 2. Materials and Methods

### 2.1. Honey Bee Colony Selection

The experiment was conducted in late spring 2019 on *A. mellifera ligustica* colonies belonging to an experimental apiary of CREA-AA located in Bologna, Italy (44°31′26.8″ N, 11°21′04.5″ E). The apiary consisted of approximately forty colonies housed in ten-frame Dadant–Blatt (DB) hives asymptomatic for the main honey bee diseases and managed according to good beekeeping practices. Fifteen of these colonies were randomly selected for the experiment based on the presence of a *N. ceranae* infection, which was assessed in a preliminary screening conducted on pools of 25 foragers that were collected from the external combs of each colony and analyzed with the *Hsp70* qPCR assay (see Section 2.5). The selected units were divided by random sampling without replacement into three experimental groups of five colonies: R (*E. sativa*), N (*B. nigra*) and CTRL– (untreated control).

### 2.2. Brassicaceae DSMs

*Brassica nigra* (L.) W.D.J. Koch and *Eruca sativa* Mill. seeds were available in the seed collection of Brassicaceae of CREA-CI (Bologna) [49]. The two crops were cultivated in the experimental open fields of CREA-CI located at Anzola (Bologna) by using low-input cultivation techniques and no pesticides. Seeds were harvested, cleaned, ground, defatted by mechanical pressure and deactivated for the MYR enzyme as described by Nanetti et al. [55]. The procedures permitted obtaining homogeneous deactivated DSMs, finally sieved at 125 μm and stored at room temperature until use. DSMs were characterized for moisture, proteins, residual oil, total GSLs and phenolic content according to Nanetti et al. [55]. The oil and protein content was expressed as the *w*/*w* percentage on dry matter (DM). The GSL amount was expressed as μmol/g of the DSM, the total phenol content—as mg gallic acid equivalents (GAE)/g of the DSM.

### 2.3. Feed Formulation

Deactivated DSMs of *B. nigra* and *E. sativa* were mixed at 4% (*w*/*w*) with sugar candy ApiCandy (Chemicals Laif, Padua, Italy) and 1% of water using an electric mixer. The sugar candy was composed of water at 3%; pH 5.5; carbohydrates at 97% (fructose, 7.5%; glucose, 8%; sucrose, >60%). The components were mixed until homogeneity, divided in aliquots of about 250 g per treated hive, wrapped in a plastic film and weighted. GSL content stability in the DSM patties was verified at the beginning of trials and after one week of storage in the hive by means of hot ethanol–water (70%) GSL extraction and GSL analysis performed according to Nanetti et al. [55]. A candy mixed only with 1% water was prepared following the same protocol (negative control, CTRL–).

### 2.4. Treatments and Colony Handling

The randomly selected colonies were experimentally fed. The treatments included two DSM patties enriched with a Brassicaceae DSM at 4% (*w*/*w*), both for *E. sativa* or *B. nigra* (R and N), and one negative control receiving candy with no DSM added (CTRL–). For each treatment, 250 g of patties were administered every week for four weeks.

The development status of the colonies was evaluated every week using the modified Liebefeld method according to Accorti (1985) and Marchetti (1985) [56,57]. Briefly, the surface of each comb side that was found covered by adult bees or with brood was visually estimated. The unit of measurement was the sixth part of the DB comb surface. The sum of the values obtained for each colony provided the total surface occupied by the bees or containing brood.

Underbasket cages were kept under the entrance to each colony throughout the experimental period to assess the worker mortality [58,59]. The cages were inspected every 3–4 days and emptied after counting the dead bees.

Twenty-five foragers and young workers were separately sampled from each colony before treatment (14 May) and after treatment (11 June). The foragers were collected after closing the hive entrance and waiting for a sufficient number of returning bees to gather. Young bees were collected from one of the internal brood combs. The samples were stored at −20 °C until analysis [60].

### 2.5. N. ceranae Infection Quantification: DNA Extraction and qPCR

Each sampled honey bee was analyzed individually after careful dissection as previously reported [55,61]. Briefly, the digestive tract from the ventriculum to the rectum was removed with tweezers and homogenized in 1 mL DNAse-free water with a TissueLyser II (Qiagen, Hilden, Germany) for 3 min at 30 Hz.

The total DNA was extracted from each homogenate using a Quick DNA Microprep Plus Kit (Zymo Research, Irvine, CA, USA) following the modified manufacturer’s instructions for solid tissue processing [62,63].

The obtained DNA extracts were analyzed by means of qPCR with primers and probes specific for *N. ceranae* designed on sequences of the *Heat-shock protein 70* (*Hsp70*) gene [63]. A total reaction volume of 15 μL was prepared using a 2x QuantiTect Probe PCR Master Mix (Qiagen, Hilden, Germany), forward and reverse primers (2 μM), forward and reverse probes (500 nM) and 3 μL DNA extract.

The standard curve was generated by amplifying the serially diluted recombinant plasmids containing the *N. ceranae*-specific DNA fragment from 100–10^9^ copies in a qPCR assay as previously reported [60]. The qPCR assay was performed on a Rotorgene Corbett 6000 (Corbett Research, Sydney, Australia) following the amplification and quantification protocols [63].

All the analyses were conducted with two technical replicates.

### 2.6. Bee Gut MYR Enzyme Extraction and Activity Assay

The gut sampling for MYR activity assay was performed one week after the fourth candy administration at the end of the trial. Ten foragers per group (N, R, C) were collected from the hives, sacrificed and dissected. On a Petri dish placed on an ice block, their midgut and hindgut were separated and grouped (*n* = 10) into different 1.5 mL tubes containing 300 μL of the protein extraction buffer (20 mM Tris HCl, 0.15 M NaCl, 5 mM EDTA (pH 8)) supplemented with protease inhibitors (cOmplete EDTA-free; Roche CustomBiotech, Mannheim, Germany) [33] that were promptly deep-frozen by immersion into liquid nitrogen and stored at −80 °C until analysis. Enzyme extraction of the bee midgut and hindgut was achieved by homogenization of thawed samples in the extraction buffer using a TissueLyser II (Qiagen, Hilden, Germany) (30 Hz, 5 × 30 s, at 4 °C). The crude extract was subsequently recovered by centrifugation at 31,500× *g* for 20 min at 4 °C, the supernatant was separately collected and the pellet was extracted again with 300 μL of the extraction buffer. The two extracts were combined and used for the MYR activity assay. The midgut extracts were concentrated 4.5 times to better detect MYR activity by ultrafiltration with Amicon Ultra filters at 10 kDa (Millipore Corporation, Billerica, MA, USA).

#### 2.6.1. MYR Activity Assay

MYR enzymatic activity in the gut extracts was qualitatively evaluated by means of GSL hydrolysis product detection using headspace solid-phase microextraction (SPME) coupled with GC–MS analysis. Extracts (10 μL) were incubated with 50 μL of 10 mM of sinigrin (allyl GSL–SIN) or glucoerucin (4-methyltiobutyl GSL–GER) standards isolated and purified (99% HPLC purity and 96% purity on weight basis) from *Brassica juncea* and *E. sativa* Mill [64] respectively, previously dissolved in 50 mM K phosphate buffer (pH 6.5). The solutions were maintained for 5 min at room temperature and under magnetic agitation in 1 mL glass vials with a polytetrafluoroethylene/silicone septa cap. SPME extraction was performed by exposing a fiber assembly 50/30 μm divinylbenzene/Carboxen/polydimethylsiloxane (StableFlex™/SS, 2 cm, Supelco, Bellefonte, PA, USA) to the vial headspace for 30 min at 25 °C. Blank samples of the buffer with the substrates but without extracts and of the buffer with extracts but without substrates were also analyzed following the same procedure. Thermal fiber desorption was achieved by insertion in the inlet of the gas chromatograph (GC) for 2 min at 250 °C, and GSL hydrolysis products analysis by GC–MS followed. A Scion GC 436 gas chromatograph coupled with a Scion SQ quadrupole mass detector (Scion Instruments NL BV, Amundsenweg, The Netherlands) and equipped with a Rtx^®^-2330 capillary column (30 m × 0.2 mm i.d., 0.25 μm film; Restek S.r.l., Milano, Italy) was used. The oven temperature was set at 60 °C for 4 min and then programmed to rise from 60 to 160 °C at 10 °C/min, hold 160 °C for 8 min, rise again from 160 °C to 260 °C at 40 °C/min and finally hold 260 °C for 7 min. The transfer line was heated to 250 °C, the ion source to 220 °C. Helium carrier gas had a flow of 1 mL min^−1^. The splitless injection mode was used. The mass spectrometer operated in the electron impact mode at 70 eV, scanning the range of 35/500 *m*/*z* in the full scan acquisition mode. Allyl isothiocyanate (AITC) and erucin ITC (4-methylthiobutyl ITC, ERITC) identification was achieved by comparing the mass spectra with the data system library (NIST 11 MS Library) and the ITC GC retention time and mass spectra with those of pure standard compounds. A commercial AITC standard (>99% purity; Sigma-Aldrich, St. Louis, MO, USA) and the ERITC standard produced and purified from the GER GSL as described in [64] (>98% *w*/*w*) were used. Response coefficients of 3.28 and 3.72 were calculated using the ratio of the slope of the calibration curves obtained from the AITC and ERITC pure standards, respectively, and the internal standard benzyl isothiocyanate (99.8% purity; Sigma-Aldrich) solutions in hexane (0.1–40 nM) analyzed under the same SPME GC–MS conditions. Extraction ion chromatograms (EIC) were also produced for a specific *m*/*z* (99 for AITC and 115 for ERITC).

### 2.7. Bee Gut Total ITC Extraction and Quantification

Gut sampling for ITC determination was performed as soon as the last candy was completely consumed by bees after the last administration at the fourth week of treatment in order to avoid possible elimination by the bee or further degradation of eventually formed ITCs in the gut. Five foragers per group, CTRL included, were sacrificed and dissected for midgut and hindgut collection in 1.5 mL empty tubes that were promptly deep-frozen in liquid nitrogen and stored at −80 °C until analysis. Three replicates from each hive were collected.

Midgut samples were extracted two times with 300 μL and 250 μL of cold pure methanol using a TissueLyser II (Qiagen, Hilden, Germany) (30 Hz, 5 × 30 s) and subsequently centrifuged at 31,500× *g* for 20 min at 4 °C according to Nanetti et al. [55]. Hindgut samples were extracted with double volumes following the same procedure. A combination of the two extracts (500 μL) was used for the cyclocondensation assay with 1,2-benzenedithiol to quantify the total ITCs as described in [50]. The cyclocondensation product, 1,3-benzodithiole-2-thione, was analyzed using a Hewlett-Packard chromatograph 1100 equipped with a diode array detector and a Zorbax SB-C18 column (150 × 4.6 mm, 3.5 μm; Agilent Technologies, Santa Clara, CA, USA) thermostated at 30 °C. Chromatography was performed at a flow rate of 1 mL/min (elution) with 1% formic acid in water (A) and methanol (B) as follows: 5 min 80% B; 3 min 100% B; 2 min 80% B; 1,3-benzodithiole-2-thione was detected monitoring the absorbance at 365 nm. An external calibration curve was generated using methanolic solutions of pure AITC and ERITC standards to obtain the cyclocondensation product for ITC quantification in the gut derived from the bees fed on N and R, respectively. The results were expressed as pmol/mg in gut tissues.

### 2.8. Honey GSL and ITC Detection

Honey samples (about 50 g) were harvested from five treated hives from each group R, N and CTRL–. Honey GSL content was determined by following the procedure described above for DSM GSL analysis (Section 2.2).

GSL hydrolysis products were qualitatively detected in honey samples by means of SPME and GC-MS analysis following Pasini et al. [65] with some modifications; 1 g of honey was 1:1 diluted with 30% NaCl (*w*/*v*) aqueous solution and incubated for 5 min at 40 °C under agitation in 10 mL glass vials closed with a screw plug pierceable septum. SPME fiber was inserted in the vial and exposed to the headspace for 20 min of extraction at 40 °C under agitation. Compound desorption took place in the GC injection port for two min at 250 °C and was followed by gas chromatographic analysis on a GC–MS Scion SQ as described above (Section 2.6.1) with some modification in the oven program: initial temperature was maintained at 40 °C for 4 min, raised to 140 °C at 10 °C/min, than to 240 °C at 40 °C/min and finally held at 240 °C for 4 min. A blank sample without honey and control honey samples free of known GSL hydrolysis compound contamination, spiked with 1 μL of purified GSL hydrolysis products, ITC (AITC, ERITC) or nitriles (allyl cyanide, ACN, or erucin nitrile, ERN), diluted in hexane, were analyzed in order to compare retention time and spectra with the detected compounds in honey from treated colonies. The extraction fiber used was coated with 75 μm Carboxen–polydimethylsiloxane film (CAR–PDMS) (Supelco, Bellefonte, PA, USA). The ACN standard was purchased form Sigma (>98% purity; Sigma–Aldrich), while ERN was produced and purified from the GER GSL as described in [66] with a purity > 95% (*w*/*w*). Extraction ion chromatograms (EIC) were also produced for a specific *m*/*z* (67.0 for ACN and 129.0 for ERN).

### 2.9. Calculations and Statistics

The cumulative worker mortality was expressed as the sum of the bees that were found dead in each underbasket cage throughout the experiment.

The coefficients 250 and 750 (empirically determined) were used to convert the surface covered with bees or containing brood into the number of, respectively, the workers populating each colony and the cells present in the respective nest.

The *N. ceranae* abundance at the individual bee level was expressed as the average of the number of copies detected in the two technical replicate analyses. Those data were used to calculate the prevalence of the infected bees in each sample.

The *N. ceranae* abundance of the bees in each sample was averaged to obtain the abundance at the colony level. The ratio between post- and pretreatment abundance was calculated to estimate the variation of abundance during the treatment.

The number of bees, amount of brood cells and *N. ceranae* abundance were compared before/after treatment in the same colonies with Student’s *t*-test for paired data.

A nonparametric statistic approach was used to analyze the other experimental results. The effect of the treatment group as a categorial factor on the dependent variables of interest was assessed with a one-way ANOVA Kruskal–Wallis H test. In case of a significant effect, the between-groups difference was checked with a bilateral pairwise multiple comparison test of mean ranks.

Gut ITC analysis results were subjected to an ANOVA employing the least significant difference (LSD) test to assess significant differences between the analyzed samples.

For all the statistics, a protection level against type I errors was set at *p* ≤ α = 0.05.

## 3. Results

### 3.1. DSM Characterization and Patty Formulation

Homogenous fine powder of DSMs of *E. sativa* and *B. nigra* defatted using food-grade mechanical seed oil extraction and deactivated for MYR content were finally characterized as reported in Table 1.

DSMs showed a good protein content and a low percentage of oil, higher for *E. sativa* DSM, in accordance with a previous work [55].

The aliphatic SIN was the main GSL in *B. nigra* DSM (>95%), while two thio-functionalized GSLs, GER and GRA, were found in *E. sativa*, accounting for 90.7% and 9.3% of the total GSL content.

The total phenol content was higher for the *B. nigra* DSM, while the *E. sativa* DSM content was comparable with that found for the same variety or other Brassicaceae DSMs used in previous works [52,55,67].

DSMs were formulated with sugar candy at 4% *w*/*w* concentration, which gave patties with a total GSL concentration of 3.99 and 5.23 μmol/g for the R and N groups, respectively. GSL stability in the formulated patties was verified after one week of storage as the maximum storage period in the hive. GSL analysis showed that 93.0% and 95.7% of the initial total GSL concentration in the R and N patties, respectively, was found at the end of the first week of treatment, demonstrating good stability of the GSLs in the formulate.

### 3.2. Bee Treatment Feeding Trials

Food consumption and bee mortality were monitored during the feeding trial with the R and N patties for up to 4 weeks.

#### 3.2.1. Palatability of DSM Patties

All the groups completely consumed the food within the early days of administration, and the control patty was consumed even quicker (within 48 h) than DSM patties. The average overall assumption was 1004.2 g of food per hive and 40.2 g of DSMs or 3962.9 and 5254.1 μmol of total GSLs for the R or N group, respectively.

#### 3.2.2. Effect of DSM Patties on Bee Survival

**Figure 1:**
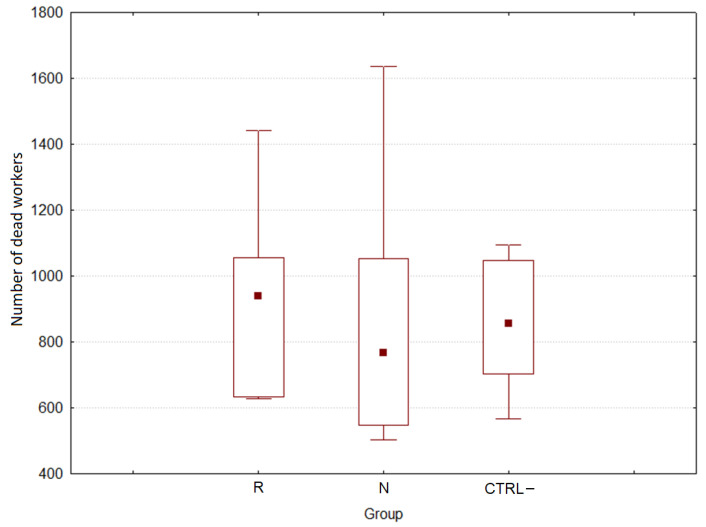
Bee workers cumulative mortality from the three groups (R: *E. sativa*, N: *B. nigra*, CTRL–: negative control) during feeding trials of 28 days.

The cumulative mortality of worker bees is reported in Figure 1.

The cumulative mortality was 898.3 ± 85.8 (s.e.) bees/colony, and no significant differences were registered between treatments and CTRL– (H(2, *n* = 15) = 0.320, *p* = 0.852).

**Figure 2:**
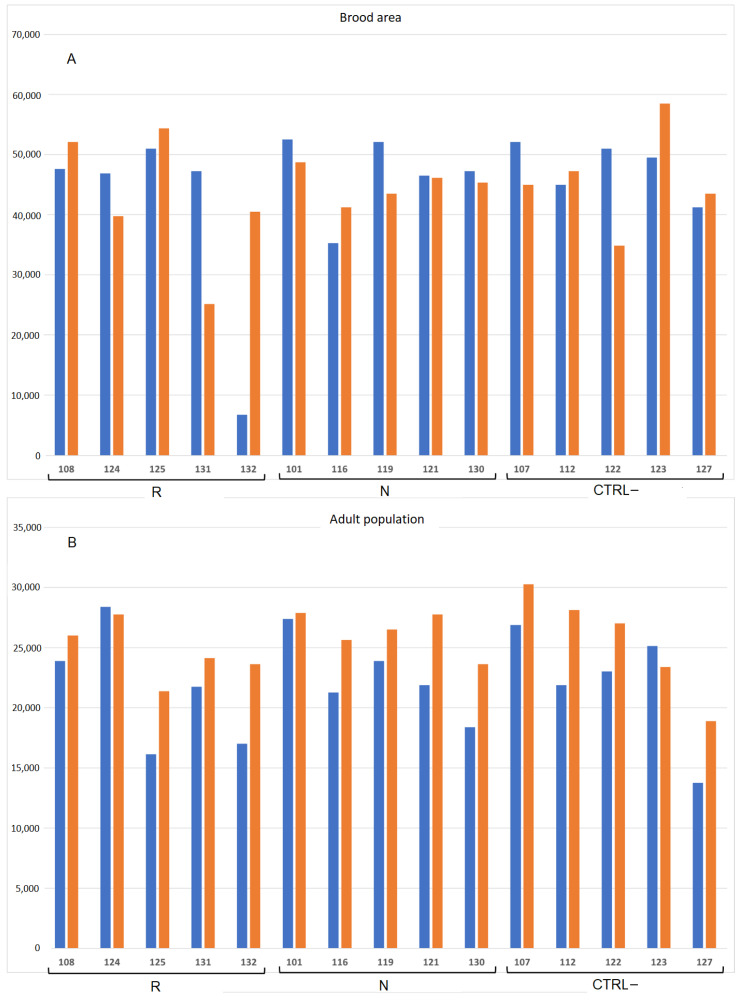
Number of brood cells (**A**) and adult bees (**B**) registered before (blue) and after treatment (orange) in the colonies (number ID) of the three groups (R: *E. sativa*, N: *B. nigra*, CTRL–: negative control).

Figure 2 and Table S1 (raw data) show the adult and the brood population before and after treatment.

Before treatments, colony population was composed of 22,033.3 ± 1096.4 adult bees and 44,800.0 ± 2963.6 brood cells. The Kruskal–Wallis test showed no significant differences between the experimental groups both for adult population (H(2, *n* = 15) = 0.196 *p* = 0.907) and brood area (H(2, *n* = 15) = 0.382, *p* = 0.826).

At the end of the trials after 4 weeks, the population of the 15 colonies was significantly larger (25,458.3 ± 765.5 adult bees) than the initial population (t(15) = −5.227, *p* < 0.000); however, the ratio between the population before and after 4 weeks was not significantly different between the groups R, N and CTRL– (H(2, *n* = 15) = 0.035, *p* = 0.983). Instead, the brood area did not change during the trial (t(15) = 0.122, *p* < 0.904).

#### 3.2.3. Effect of DSM Patties on *N. ceranae*

In foragers, no significant difference was found in the pre-treatment *N. ceranae* infection level (H(2, *n* = 15) = 4.500, *p* = 0.105) (Table 2). Considering the experimental colonies in general, the *N. ceranae* abundance did not significantly change in the pre-/post-treatment interval (t(14) = 0.255; *p* = 0.803).

However, the post-treatment samples showed significantly different *N. ceranae* abundance (H(2, *n* = 15) = 9.500, *p* = 0.009). In particular, in the groups R and N, significantly fewer *N. ceranae* copies were detected compared to CTRL– (z = 2.475, *p* = 0.040; and z = 2.828, *p* = 0.014, respectively), but no significant difference was found between the treated groups (z = 0.353, *p* = 1.000).

Contrary to the foragers, the pre-treatment *N. ceranae* abundance in house bees was significantly different between the groups (H(2, *n* = 15) = 6.500, *p* = 0.039), with milder infections detected in the R colonies than in the CTRL– colonies (z = 2.475, *p* = 0.040). Overall, the *N. ceranae* abundance was found significantly decreased in the post-treatment samples compared to the pre-treatment values (t(14) = 5.000; *p* = 0.000).

The post-treatment abundance was significantly influenced by the treatment groups (H(2, *n* = 15) = 10.500, *p* = 0.005), with lower values in the R samples than in the CTRL– ones (z = 3.182, *p* = 0.004).

**Figure 3:**
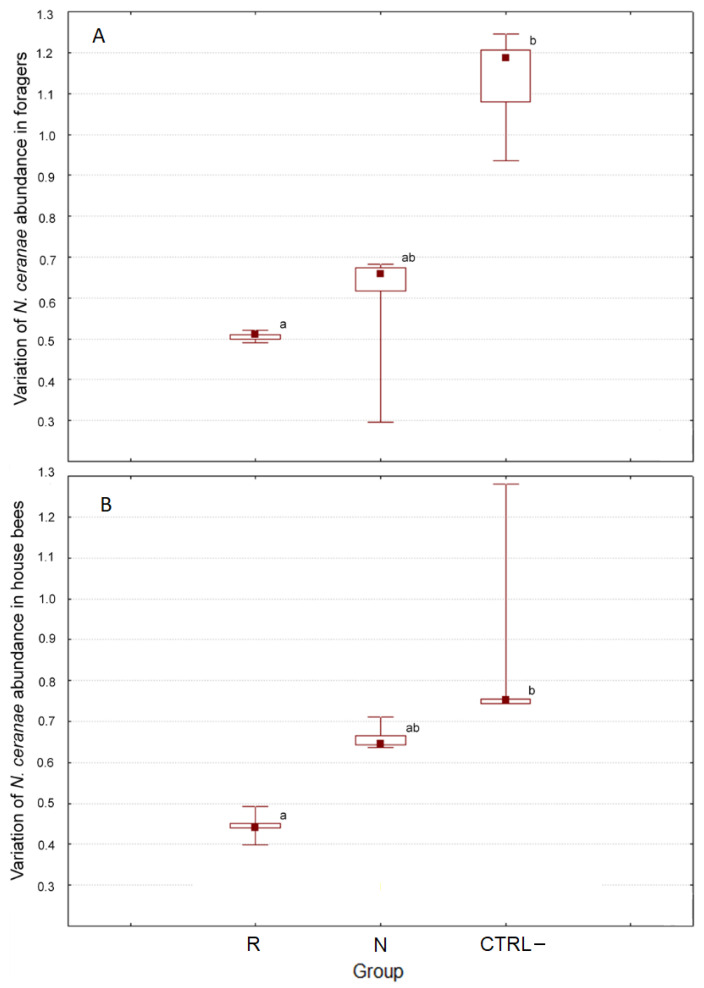
Post-/pretreatment variation of *N. ceranae* abundance in foragers (**A**) and house bees (**B**). Different letters indicate significant differences (Kruskal–Wallis H test, *p* = 0.05).

Figure 3 shows the variations in the *N. ceranae* abundance in the different groups in the pre-/post-treatment interval that was significantly influenced by the group in both foragers (H(2, *n* = 15) = 10.500, *p* = 0.005) and house bees (H(2, *n* = 15) = 12.500, *p* = 0.002).

In detail, the variation of *N. ceranae* abundance in the foragers from the CTRL– colonies (median = 1.19) was significantly different (z = 3.182, *p* = 0.004) compared to the samples from the R group (median = 0.51). The corresponding variation in the N colonies (median = 0.66) was not significantly different from that in the other groups (Figure 3A). Similarly, the variation of abundance in house bees from the CTRL– group (median = 0.75) was significantly different (z = 3.536, *p* = 0.001) compared to the samples from the R group (median = 0.44), but the variation in the N group (median = 0.64) did not significantly differ compared to that in the other groups (Figure 3B).

The prevalence of infected bees in the samples was always 100% irrespective of treatment (R, N, CTRL–), age (foragers, house bees) and period of sampling (before and after treatment).

### 3.3. MYR-Like Enzymatic Activity in the Bee Gut

In order to investigate the bee metabolism of GSLs consumed with DSM-rich diets, β-thioglucosidase activity was qualitatively searched in bee gut extracts obtained from the bees fed on N and R patties and bees fed on candy alone, without the addition of DSM (CTRL–). The extracts were incubated with pure SIN or GER as the substrate, in a buffer at pH 6.5 and 25 °C, and the headspace was analyzed for volatile GSL hydrolysis products detection by SPME coupled with GC–MS.

**Figure 4:**
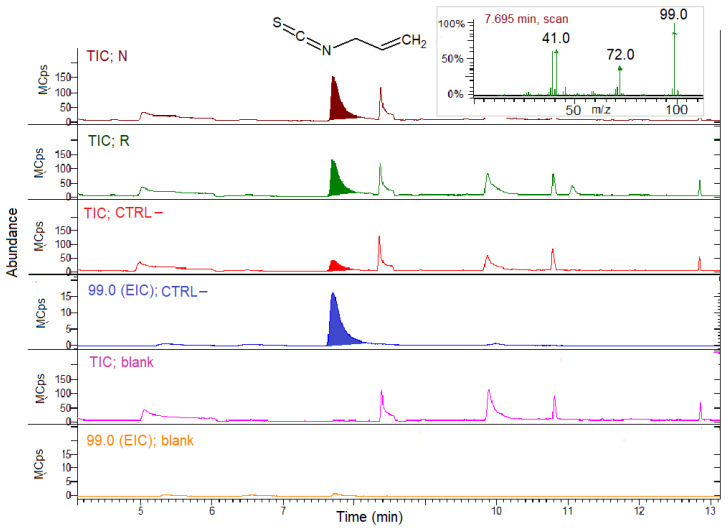
SPME GC–MS analysis of concentrated midgut extracts from the bees fed on N, R and CTRL– patties for myrosinase activity detection with sinigrin (SIN) as the substrate. Blank sample chromatograms represent the analysis of the buffer with SIN, but without the gut extract. TIC, total ion chromatogram; EIC; extracted ion chromatogram (99.0 *m*/*z* for allyl isothiocyanate, AITC). Inset: AITC MS spectrum.

The TIC and EIC profiles of the N, R, CTRL– concentrated midgut extracts and the blank sample of buffer incubated with SIN without the extracts are reported in Figure 4.

The TIC showed the occurrence of the volatile AITC peak at 7.7 min in all the samples, derived by the hydrolysis in situ of the SIN substrate in the presence of the extracts. The EIC for 99 *m*/*z* confirmed the AITC presence in all the groups, the bees fed on R or N, and in CTRL–, too. The EIC for CTRL– was reported as an example in Figure 4. Instead, no AITC was detected in the samples where the substrate SIN was incubated in the buffer in the absence of the gut extract (blank sample).

**Figure 5:**
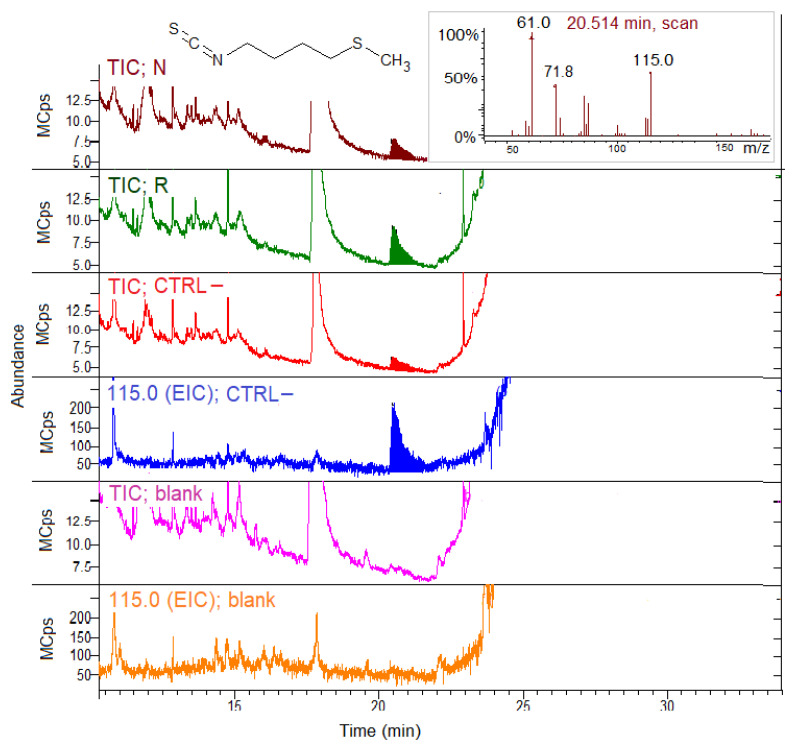
SPME GC–MS analysis of the concentrated midgut extracts from the bees fed on N, R and CTRL– patties for myrosinase activity detection with glucoerucin (GER) as the substrate. The blank sample chromatograms represent analysis of the buffer with GER, but without gut extracts. The chromatograms were expanded to point out the erucin isothiocyanate (ERITC) peak. TIC, total ion chromatogram; EIC; extracted ion chromatogram (115.0 *m*/*z* for ERITC). Inset: ERITC MS spectrum.

Figure 5 shows the expanded TIC and EIC of the N, R, CTRL– concentrated midgut extracts and the blank sample incubated with GER.

TIC shows the in situ ERITC production at 20.5 min from all the group extracts incubated with GER. No ERITC was produced from GER in the absence of extracts (blank sample). The chromatograms were expanded in order to point out the ERITC peak, which was quite small. The EIC for 115 *m*/*z* confirmed the ERITC presence in the N, R and CTRL– groups, but not in the blank sample. The CTRL– and blank EIC were reported as an example in Figure 5. Despite the similar SPME GC–MS response factors (see Section 2.6.1), the ERITC peak areas (Figure 5) were considerably lower compared to the AITC areas (Figure 4) obtained incubating the extracts with two substrates at the same analytical conditions.

ITCs were the major products formed in situ by incubating the concentrated midgut extracts with both SIN or GER, indicating the presence of MYR-like enzyme activity. Other hydrolysis products, such as nitriles, were not found even by extracting specific *m*/*z* for nitriles. The samples obtained by incubating extracts in the buffer without the substrates did not show the presence of GSL hydrolysis products (not shown).

The same qualitative results were also obtained with hindgut extracts (not shown), revealing MYR activity both for SIN or GER as substrates.

### 3.4. Total ITC Gut Analysis

Midguts and hindguts from the bees fed on N and R patties were analyzed for the total ITC content by means of the cyclocondensation assay in order to see if the detected MYR activity in the bee gut actually hydrolyzed GSLs consumed with DSM-rich diets in vivo. Total ITCs were detected as the derivatization product 1,2-benzenedithiol-2-thione formed from AITC and ERITC plus sulforaphane in the N and R samples, respectively. ITC adducts, possibly formed by ITC reaction with amino acids or protein in the medium (namely dithiocarbamates), also give the same derivatization product [68] and were thus included in the quantification. The results are reported in Table 3.

ITCs were found and quantified in the different gut tissues of the bees fed on the two Brassicaceae DSMs, while no ITCs were found in the CTRL– samples. The quantity of ITCs found in the hindgut was significantly higher than in the midgut for both R and N groups, while the ITC amount from N seemed slightly lower than that found in R, but the difference was not significant.

The cyclocondensation assay allowed detecting ITCs in the gut tissues, even if they were not revealed by the SPME GC–MS analysis in the sample where the extracts were incubated in the buffer without a substrate. This could be due to different assay sensibility or indicate that the majority of detected ITCs were in the form of adducts only detectable with the cyclocondensation assay. The SMPE GC–MS analysis, in fact, could only detect ITCs in the free volatile form.

### 3.5. GSLs and GSL Hydrolysis Products in Honey

Honey collected from hives of the R, N and CTRL– groups was analyzed for GSL content, and the results are reported in Table 4.

SIN was traced in samples of honey produced by the bees fed on N patties, while GRA, the oxidation product of GER, which was the main GSL originally present in the *E. sativa* DSM, was detected in honey produced by the bees fed on R patties. Their content expressed on weight basis corresponded to 15.9 mg/kg and 5.24 mg/kg for SIN and GER, respectively.

**Figure 6:**
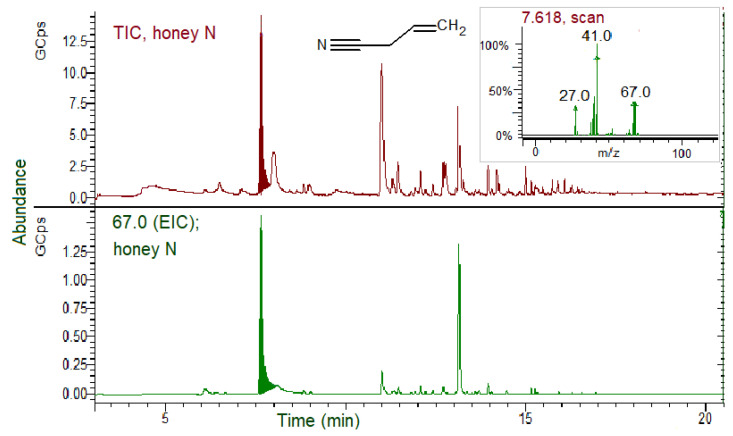
SPME GC–MS analysis of honey produced by bees fed on *B. nigra* patties (N group) for 28 days. TIC, total ion chromatogram; EIC; extracted ion chromatogram (67.0 *m*/*z* for allyl cyanide, ACN). Inset: ACN MS spectrum.

**Figure 7:**
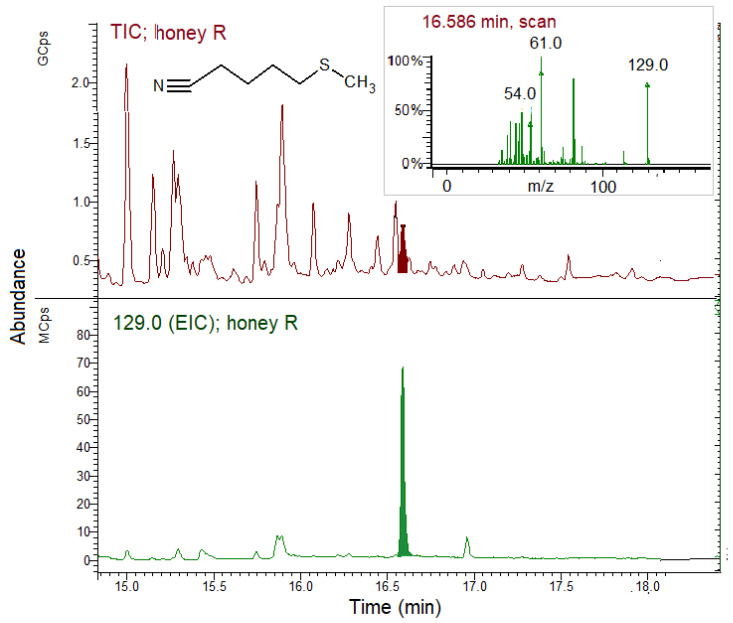
SPME GC–MS analysis of honey produced by the bees fed on *E. sativa* patties (R group) for 28 days. The chromatograms were expanded to point out the ERN peak. TIC, total ion chromatogram; EIC; extracted ion chromatogram (129.0 *m*/*z* for erucin nitrile, ERN). Inset: ERN MS spectrum.

A qualitative evaluation of the honey volatile fraction by SPME GC–MS analysis instead revealed the presence of GSL hydrolysis products, this time recognized as nitriles derived from the original GSLs, while ITCs were not detected under the analytical conditions. Figure 6 and Figure 7 show the TIC and EIC of the honey collected from one hive each of the N and R groups, respectively.

In Figure 6, a high TIC peak of ACN is evidenced in honey produced by bees from the N group, and the relative EIC for the specific ion confirmed its identification. The ACN relative percentage area (% of the total volatile content) estimated from the TIC peaks areas was 24.2%. No AITC was detected. All five samples of the honey collected from the N hive replicates showed nitrile presence, giving similar chromatograms.

Figure 7 shows the expanded TIC and EIC of the volatile fraction of honey harvested from the R group hive. Traces of ERN were found in only one of the five honey samples produced by the R groups and gave a peak with a low relative area of 0.41%. Neither nitriles nor ITCs were found in the other replicates.

No GSL hydrolysis products were found in honey derived from the CTRL– samples.

## 4. Discussion

DSMs of *B. nigra* and *E. sativa* were selected for feed treatment against *N. ceranae* infections in field trials as their use previously showed promising results in infection containment in bees grown in cages in laboratory conditions at the concentrations of 2% and 4% in sugar candy [55]. The higher DSM concentration of 4% was chosen for field treatments described in this work, even for *B. nigra*, although it showed at this concentration some bee toxicity in laboratory trials. DSMs were particularly concentrated in GSLs, SIN in *B. nigra* and GER and GRA in *E. sativa*, the precursors of bioactive compounds mainly responsible for their biological activity. The structurally related GER and GRA and their interconvertible hydrolysis products erucin and sulforaphane in particular are the most studied ITCs for their protective effects against cancer in several tissues [64,69]. Anyway, other components such as proteins and phenols could contribute to DSM nutritional and nutraceutical properties [47].

The administrated DSM patties did not significantly affect the colony development. Colony population and worker mortality were homogeneous among the groups throughout the experimental period. This corroborates both correctness of the randomization method that was used and treatment tolerability at the individual and colony level. The significant increase in both adult population and brood area that occurred in the treatment period reflects the normal dynamic of the spring colony development [70,71].

High GSL concentrations were found associated with high bee mortality in no-choice feeding experiments conducted on caged workers [54,55]. Similar effects were not confirmed in the managed honey bee colonies used in this experiment. Fully developed colonies definitely represent a situation not easily comparable to small groups of bees of the same age confined in cages and reared in an incubator. The dose administered to fully developed colonies is shared among a large number of the members of the superorganismic group by trophallactic exchanges [72] and diluted with other feeding sources coming from the available floral resources or from nest stores.

The treatments did not influence the *N. ceranae* prevalence of the infected bees, all of which were found infected in both pre- and post-treatment samples of foragers and house bees. Throughout the experiment, significant variations in *N. ceranae* abundance were detected. In particular, the treatment with patties incorporating DSM from *E. sativa* at the concentration of 4% appeared to significantly decrease the number of *N. ceranae* copies in both foragers and house bees. Our results confirm the effects of the two DSMs recorded in previous laboratory bioassays on this infection [55], even though that trial highlighted higher effect against *N. ceranae* with DSM patties supplemented with 4% of *B. nigra*.

ITCs were previously found and quantified in the midguts and hindguts of the bees fed on *B. nigra* and *E. sativa* patties in laboratory experiments, indicating that consumed GSLs from DSMs were metabolized into ITCs [55]. In this work, guts of the bees similarly treated in field trials were extracted and in vitro assays were performed in order to search for MYR activity in this tissue. A qualitative investigation of MYR activity gut presence was thus pursued by incubating bee gut extracts with pure GSLs as MYR substrates and showing the in situ hydrolysis of GSLs by means of hydrolysis product detection. SPME followed by GC–MS analysis was used as a simple and rapid technique for sampling volatile GSL hydrolysis products produced at low concentration in the headspace [73]. ITCs were the main hydrolysis products formed in vitro at pH 6.5 and 25 °C using both midgut and hindgut extracts derived from bees of the N, R groups, but also of the CTRL– group, thus confirming the presence of an endogenous MYR-like activity in bees capable of metabolizing GSLs into ITCs. Previous studies found MYR activities in the gut of the larvae of the leaf beetle *Phaedon cochleariae* (Coleoptera: Chrysomelidae) or the sawfly *Athalia rosae* (Hymenoptera: Tenthredinidae) fed on Brassicaceae plants, but they ascribed the activity to the ingested plant tissue endogenous MYR [34,74]. Instead, in this work, DSMs were treated in an autoclave for MYR deactivation, so MYR activity can be specifically assigned to the bee gut extracts. AITC was detected by means of incubation of all the extracts with SIN, while ERITC was formed from GER as the substrate. No other hydrolysis products were produced in vitro at the assay conditions; however, other breakdown products could be formed in vivo depending on cellular conditions or the presence of MYR co-factors. The pH, for instance, which increases along the bee midgut up to 7.0 and decreases toward the ileum and rectum to 5.2 [75], can possibly influence MYR activity. Interestingly, the presence of MYR activity in the CTRL– samples indicated that a constitutive MYR-like enzyme was even present in the bees not treated with Brassicaceae DSMs. A possible induction of this activity by a DSM-rich diet cannot be ruled out. In humans, for instance, GSL hydrolysis by gut microbiota is poor unless it is previously exposed for days to GSL-containing food [76] and can be significantly different among individuals on the basis of their health status and microbiota composition [77,78].

ITCs were also found and quantified as total ITCs in the free form or as adducts in the midgut and hindgut collected from the bees fed on R and N patties. The results on MYR activity discussed above justify the gut presence of ITCs formed as a consequence of GSL assumption. ITC gut concentration was found higher in the hindgut with respect to the midgut, confirming previous results obtained within laboratory trials [55]. Anyway, ITC gut concentrations had lower values with respect to laboratory trials [55], where DSM patties were administered to bees at a half (2%) concentration of that used in this work. These differences could be justified by considering that colony bees in open-field trials are free to feed on other nutriments apart from DSM patties. Furthermore, caged bees did not defecate during laboratory trials and did not excrete the ITCs, which in turn could have been accumulated in the gut.

MYR enzymes have not been previously identified in bees, but they were isolated and characterized in other brassica specialist insects, such as *Phyllotreta striolata* flea beetles [33]. On the other hand, bees have endogenous β-glucosidase enzymes used for carbohydrate breakdown, which are produced in the head, accumulate in the midgut and persist in the hindgut [79,80]. Therefore, MYR, a β-thioglucosidase, could be part of this enzymatic equipment. Otherwise, membrane-bound glucoside hydrolase activities were found in bee gut microbiota which is most represented in the hindgut [80]. Few bacteria, in fact, colonize the bee crop and midgut, while the hindgut harbors the greatest abundance of microbiota [75]. Indeed, MYR activity detected in the bee gut could come from bee microbiota as animal and human gut microbiota showed to carry on GSL metabolism into ITCs and other hydrolysis products [81]. This hypothesis could explain the higher amount of ITCs found in the hindgut with respect to the midgut even if both host and bacterial MYR could have contributed in a different way in the two intestinal tracts to bee GSL hydrolysis. Those aspects are still under investigation.

Finally, honey produced by the bees fed on DSMs was analyzed for GSL and GSL hydrolysis product content. Honey showed to contain GSLs, SIN and GRA for the N and R groups, respectively, while GER was not detected. SIN was probably derived from *B. nigra* DSM as the prevalent GSL. GRA could be derived both from the *E. sativa* DSM, even if its GRA content was only 9.2% of the total GSLs, and from DSM GER as a bee metabolism product or as a non-enzymatic degradation product formed after GER bee release in honey. To date, the GSL presence in honey has been poorly investigated. GSLs were firstly detected and characterized in honey derived from the herbaceous Brassicaceae plant *Diplotaxis tenuifolia* and their presence was proposed for a potential use as botanical biomarkers or as a parameter for honey freshness quality [81]. Zhang et al. found GSLs in honey of *B. napus* [82], while Are et al. identified GSLs in honey from different botanical origins [83] but at much lower concentration levels (<25 μg/kg) than the R and N honey samples collected in this work. Due to the well-known beneficial properties of GSLs as precursors of healthy compounds, the richness in GSL could represent a precious added value to honey as food for human consumption. GRA in particular could be metabolized by the human gut microbiota in sulforaphane, which is the most studied ITC for its protective activity against a variety of cancers, cardiovascular, neurodegenerative diseases and diabetes [84].

The qualitative SPME GC–MS analysis of the volatile fraction of honey was also performed and revealed the presence of nitriles. ACN, probably derived from SIN hydrolysis, was particularly evident in the analysis of honey harvested from the N group, and it was one of the most represented volatiles in the honey profile. Instead, traces of ERN, probably derived from GER hydrolysis, was detected as a small peak from the analysis of one sample from the R group. Nitrile compounds were rarely searched in honey. They were previously identified in *Taraxacum*-labeled honeys, and authors attributed the contribution to honey composition of other plants, such as Brassicaceae [85]. The finding of nitriles possibly indicates the presence of MYR activity in the bee enzymatic kit for honey production. Nitriles are produced in plants by the action of MYR in the presence of low pH (<6.5), Fe(II) ions or nitrile specifier proteins (NSP) (found in specialist insects, too) [86]. Pontoh et al. found that β-glucosidases isolated from the bee hypopharyngeal glands, honey sac and ventriculus were the same enzymes [79]. If MYR from different bee organs were the same, other factors should have led to different products, such as pH, which is quite low in honey (between 3.5 and 5.5), and could have determined the nitrile formation. Nevertheless, other enzymes could have been involved, similarly to bacteria, where nitriles are often formed as a result of GSL desulfation by a sulfatase and subsequent hydrolysis of desulfo-GSL [35]. Regarding the possible contribution of nitriles to honey health properties, it has to be noted that nitrile bioactivity is generally considered less potent than that of the corresponding ITCs [87,88]. ACN and sulforaphane nitrile, which probably bear similar bioactivity compared to ERN [44], showed induction of antioxidant/phase II enzymes [88,89], which could contribute to the beneficial effect of the GSL-rich honey. As far as we know, this is the first time that GSLs and hydrolysis products of GSL were found in honey as a consequence of bee feeding with a diet rich in Brassicaceae plant products containing high amount of GSLs.

## 5. Conclusions

The results of this work obtained by testing *B. nigra* and *E. sativa* DSMs against natural *N. ceranae* infections in managed honey bee colonies showed inhibitory activity of DSMs, substantially confirming the outcomes of previous laboratory tests [55]. However, contrary to those trials, *E. sativa* seemed to perform better than *B. nigra* in field trials. Nevertheless, the prevalence did not change in the pre-/post-treatment period, and a mild effect on abundance was measured. For these reasons, other experiments are currently underway to achieve sufficient efficacy in containing the pathogen in order to obtain a bio-product based on plant matrices of practical and effective use in the beekeeping sector. Taken together, the experiments above indicate an avenue towards the use of GSLs in the control of *N. ceranae* infections affecting honey bees, but also show result instability, which suggests the need for further optimization. Despite the high prevalence of infected bees, the *N. ceranae* abundance in our colonies was undeniably low, which highlights the need to also test the treatments with fully developed infections. Certainly, full colony size and increasing development implied sharing the administered patties among a high number of members, which reduced the dose that individual bees received by food exchange. Further experiments are needed as the dose, concentration and timing of the treatments still require modifications, aiming to reach the optimal tolerability/efficacy tradeoff under the different environmental conditions. Finally, the study showed for the first time, to the best of our knowledge, the detection of MYR-like enzyme activity capable of hydrolyzing consumed GSLs into bioactive ITCs in the bee gut. Research aiming at deepening this finding in order to better understand the bee GSL metabolism pattern and the MYR origin and function would also be useful to optimize bee treatment conditions. Furthermore, the use of DSMs containing GSLs represents a promising alternative to fumagillin as it would greatly overcome the problem of toxic bee product residues encountered with antibiotic treatment. Indeed, a DSM-rich diet would allow obtaining added-value honey enriched with health-promoting compounds, such as GSLs and their hydrolysis products.

## Figures and Tables

**Table 1:** Chemical characterization of *E. sativa* and *B. nigra* DSMs. Mean values ± standard deviation (*n* = 3) are shown. GSL common variable side chain’s (R) chemical structure is also indicated, where X represents the GSL S-glucopyranosyl thiohydroximate moiety. GER, GRA and SIN indicate the GSL glucoerucin, glucoraphanin and sinigrin, respectively.

	*E. sativa* DSM	*B. nigra* DSM
Moisture (%)	5.9 ± 0.1	6.0 ± 0.1
Protein (%)	35	43.8
Oil (%)	17.9 ± 0.2	8.5 ± 0.2
GSL1 (μmol/g) side chain (R1)	GER 90.5 ± 1.9 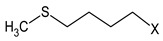	SIN 130.7 ± 4.3 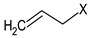
GSL2 (μmol/g) side chain (R2)	GRA 9.3 ± 0.4 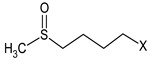	
Phenols (mg GAE/g)	7.8 ± 0.7 b	9.5 ± 0.9 a

**Table 2:** *N. ceranae* abundance (average ± standard error, standard deviation) registered in the pre- and post-treatment samples from the different groups of treatment.

		R	N	CTRL–	All Groups
Foragers	PRE	213.00 ± 3.31	199.65 ± 37.01	1284.35 ± 297.34	565.67 ± 164.32
		7.40	82.75	664.88	636.40
	POST	107.79 ± 1.92	106.17 ±1.59	1448.35 ± 723.73	555.10 ± 196.32
		4.30	3.56	323.88	760.35
House bees	PRE	202.36 ± 8.33	264.30 ± 4.67	1105.27 ± 245.39	523.98 ± 133.63
		18.63	10.45	548.71	517.55
	POST	89.62 ± 3.08	174.51 ± 4.83	840.86 ±170.56	368.33 ± 104.09
		6.88	10.79	381.38	403.12

**Table 3:** The total ITC content of midgut and hindgut tissues of the bees fed on R and N for 28 days. ITC content is expressed as pmol/mg of the gut tissue. Mean values (*n* = 5) are shown. Statistical differences between the ITC content means are indicated by different lowercase letters (*p* < 0.05, LSD test).

DSM Patty	ITC Content (pmol/mg)	
	Midgut	Hindgut
R	38.5 bc	102.9 a
N	8.3 c	80.4 ab

**Table 4:** Glucosinolate (GSL) content of honey produced by the bees fed on R and N for 28 days. GSL content is expressed as nmol/g of honey. Mean values ± standard deviation (*n* = 5) are shown.

Honey	GSL Content (nmol/g)	
	SIN	GRA
R	–	12 ± 3
N	40 ± 20	–

## Data Availability

MDPI Research Data Policies.

## Supplementary Materials

The following are available online at www.mdpi.com/article/10.3390/biom11111657/s1, Table S1: Number of brood cells and adult bees registered before and after treatment in the colonies (number ID) of the three groups (R: *E. sativa*, N: *B. nigra*, CTRL–: negative control).
