# Supplementary material for: Glucosinolate Bioactivation by Apis mellifera Workers and Its Impact on Nosema ceranae Infection at the Colony Level
Source: Biomolecules. 2021 Nov 8;11(11):1657. doi: 10.3390/biom11111657 (PMC8615805; doi:10.3390/biom11111657)
Supplement: Supplementary file 1 [file biomolecules-11-01657-s001.zip › biomolecules-1430145-supplementary.pdf]

Supplementary Materials

Table S1. Number of brood cells and adult bees registered pre- and post-treatment in the colonies (number ID) of the three groups (R: *E. sativa*, N: *B. nigra*, CTRL–: negative control). Raw data of Figure 2.

Column Number ID	Treatment Groups	Brood PRE	Brood POST	Adult PRE	Adult POST
108	R	47,625	52,125	23,875	26,000
124	R	46,875	39,750	28,375	27,750
125	R	51,000	54,375	16,125	21,375
131	R	47,250	25,125	21,750	24,125
132	R	6750	40,500	17,000	23,625
101	N	52,500	48,750	27,375	27,875
116	N	35,250	41,250	21,250	25,625
119	N	52,125	43,500	23,875	26,500
121	N	46,500	46,125	21,875	27,750
130	N	47,250	45,375	18,375	23,625
107	CTRL–	52,125	45,000	26,875	30,250
112	CTRL–	45,000	47,250	21,875	28,125
122	CTRL–	51,000	34,875	23,000	27,000
123	CTRL–	49,500	58,500	25,125	23,375
127	CTRL–	41,250	43,500	13,750	18,875
